# Knowing Ourselves Together: The Cultural Origins of Metacognition

**DOI:** 10.1016/j.tics.2020.02.007

**Published:** 2020-05

**Authors:** Cecilia Heyes, Dan Bang, Nicholas Shea, Christopher D. Frith, Stephen M. Fleming

**Affiliations:** 1 All Souls College, University of Oxford, High Street, Oxford OX1 4AL, UK; 2 Department of Experimental Psychology, University of Oxford, South Parks Road, Oxford, OX1 3UD, UK; 3 Wellcome Centre for Human Neuroimaging, University College London, 12 Queen Square, London WC1N 3BG, UK; 4 Institute of Philosophy, Senate House, Malet Street, London WC1E 7HU, UK; 5 Faculty of Philosophy, University of Oxford, Woodstock Road, Oxford OX2 6GG, UK; 6 Max Planck University College London Centre for Computational Psychiatry and Ageing Research, University College London, London WC1B 5EH, UK; 7 Department of Experimental Psychology, University College London, 26 Bedford Way, London, WC1H 0AP, UK

**Keywords:** confidence, cultural evolution, cultural learning, metacognition

## Abstract

Metacognition – the ability to represent, monitor and control ongoing cognitive processes – helps us perform many tasks, both when acting alone and when working with others. While metacognition is adaptive, and found in other animals, we should not assume that all human forms of metacognition are gene-based adaptations. Instead, some forms may have a social origin, including the discrimination, interpretation, and broadcasting of metacognitive representations. There is evidence that each of these abilities depends on cultural learning and therefore that cultural selection might shape human metacognition. The cultural origins hypothesis is a plausible and testable alternative that directs us towards a substantial new programme of research.

## Highlights

Human metacognition involves discrimination, interpretation, and broadcasting of subtle cues indicating the rightness of ongoing thought and behaviour.
We propose that human metacognition is made fit for purpose by cultural evolution rather than genetic evolution.
In particular, we present evidence that the effective discrimination, interpretation, and broadcasting of metacognitive cues depends on cultural learning.
The cultural origins hypothesis advances a programme of research on the development of metacognition, cultural variation, individual differences, and cross-species comparisons.

## Where Does Metacognition Come From?

How do cognitive mechanisms become fit for purpose? They are all complex products of nature and nurture, but who or what designs the features that enable cognitive processes to do their jobs? How come visual systems can see, learning mechanisms can learn, and reasoning processes can reason?

In many cases, **gene-based selection** (see Glossary) leads the design team. The visual system can see primarily because it has been honed by natural selection over biological generations. Variant systems were genetically inherited, and, through differential reproduction, those that were better at processing visual information proliferated while the others died out. In some cases, **intentional design** is also involved [1]. The cognitive mechanisms enabling you to read these words were designed in part by educationalists. The people who teach us to read, and designers of literacy programmes, make new cognitive mechanisms from old parts. With foresight and deliberation, they turn mechanisms that were designed by genetic evolution for small object recognition into a cognitive system for reading [2].

For some cognitive mechanisms, **cultural selection** is a third member of the design team, alongside genetic evolution and intentional design. Recent evidence suggests that a range of cognitive mechanisms, including imitation and **mindreading** (or theory of mind), have been shaped by a cultural selection process analogous to gene-based selection [3., 4., 5., 6., 7.]. In this cultural evolutionary process, variants arise in individual development, rather than by genetic mutation, and are inherited via social interaction rather than DNA. Good variants are culturally learned (e.g., copied) by more agents, but, unlike intentional design, this need not be because the teachers or the learners understand what makes them good.

In this opinion article, we suggest that an important kind of **metacognition** has been made fit for purpose primarily by the latter two members of the team – intentional design and cultural evolution – rather than genetic selection. Here, we focus on the role of cultural evolution. We survey evidence that **explicit metacognition** (Box 1) is social in origin, and we outline an empirical programme that would allow the cultural origins hypothesis to be further developed and tested. First, however, we outline the many functions of metacognition in individual and group decision-making.

Box 1 **Explicit Metacognition**

In this article we are concerned with what we call explicit metacognition. A representation is explicit, in our sense, when it is conscious and represented in working memory so that it can be used by processes of cognitive control. Thus, a hallmark of explicit metacognition is that it is sensitive to concurrent processing load. Humans typically communicate explicit metacognitive representations verbally. For example, we can tell others when we are uncertain about what we have seen. However, we can also communicate nonverbally about our explicit metacognitive states; and it is an open question whether language is necessary for an individual to have the capacity for explicit metacognition.

Metacognition also operates in implicit processes that are automatic and relatively insensitive to cognitive load. The contrast between explicit and implicit metacognition can be seen in research on error monitoring in skilled typists [130]. Automatic monitoring processes make skilled typists fractionally slower on the next keystroke after they have made an error. Explicit metacognition, by contrast, allows the typist to report that they have made an error. The factors that affect implicit and explicit metacognition in these scenarios are experimentally dissociable.

Metacognition is sometimes assumed to require consciousness, but here we adopt the more liberal definition that does not presuppose that metacognitive processes are conscious. So, a nonconscious representation or evaluation of a cognitive state or process can count as metacognitive. Explicit metacognition does require consciousness but note that our usage does not make explicit synonymous with conscious (which is another common usage). Our use is more restrictive. It excludes automatic metacognitive processes that do not depend on working memory and are insensitive to cognitive load, even if they involve conscious states like feelings of fluency.

We can further distinguish two ways in which a metacognitive assessment of a decision can be computed [48]. First-order confidence is based wholly on the state or states used to take the decision itself. Second-order confidence is computed by a separate system and considers further factors (see Figure 2 in main text). Explicit metacognition is typically the result of a second-order computation.

Alt-text: Box 1

## Metacognition Has Intrapersonal and Suprapersonal Functions

Explicit metacognition uses **conscious** representations in working memory to monitor or evaluate – and often to control – cognitive states and processes. Explicit metacognition (here metacognition, when not qualified) is sensitive to cognitive load, and is typically slow, deliberate, and verbally reportable [8,9]. It yields feelings of knowing and confidence judgements, allowing us to think and report ‘I’m sure’ and ‘I’m not so sure’ about our perceptions, memories, and decisions. The adoption of frameworks inherited from psychophysics and signal detection theory has made possible the objective measurement of metacognitive ability in laboratory tasks, by assessing the bias and sensitivity of judgments of confidence in relation to task performance [10]. Metacognitive representations allow information captured by specialised sensorimotor processes to be accessed by other processes in the same agent and by the cognitive systems of other agents – it has both intrapersonal and suprapersonal control functions [9].

Metacognition contributes to effective intrapersonal decision-making in a range of contexts. For instance, it helps ensure the smooth operation of ongoing thought and behaviour, by helping us recognise our errors [11], regulate deployment of executive functions [12,13], and detect lapses of attention [14]. It also enables cognitive offloading – the use of physical actions such as tilting the head, making notes, and finger counting – to alter the information processing requirements of a task to reduce cognitive demand [15,16]. In educational settings, metacognition regulates study time, and thereby enables children and adults to learn more from reading texts [17., 18., 19.], which in turn may contribute to the development of general intelligence [20]. Accordingly, failures of metacognition may lead to maladaptive decision-making: people who are overconfident of their knowledge about information security (a positive **metacognitive bias**) are more likely to take risks when using the internet [21], and people with weaker **metacognitive sensitivity** are more likely to hold radical beliefs at both ends of the political spectrum [22,23].

Metacognition also plays a central role in suprapersonal decision-making [9,24,25]. It not only enables individuals to monitor their own cognitive processes, but it also enables broadcast and sharing of otherwise private mental states with others. Cognitive offloading often involves depositing information with, or soliciting information from, other agents [15,26]. When people are making perceptual decisions together, ‘two heads are better than one’ when each person communicates accurate metacognitive representations about their judgements [27., 28., 29., 30.]. Jurors use witness confidence and other metacognitive representations (e.g., calibration of confidence relative to accuracy) in deciding whether to trust witness testimony [31]. When coordinating complex actions in team sports, people use metacognitive representations to decide the contribution of each team member [32,33].

The suprapersonal functions of metacognition make it plausible, from an engineering perspective, that metacognition has been shaped by cultural selection. The benefits of enhanced metacognitive skills accrue, not only to the owner of the skills, but also to other members of the social group with whom they make decisions and coordinate action. Consequently, it is in the interests of a person with enhanced metacognitive skills to teach those skills, deliberately or inadvertently, to others in their group, and there is reason to expect more skilled individuals to be more effective teachers – a condition for cultural selection.

## The Cultural Origins Hypothesis

In comparison to this focus on the functions of metacognition, there has been little enquiry about its origins – about the design team that enables metacognition to fulfil its intra- and suprapersonal roles. Researchers tend to assume that genetic evolution has played a major part in making metacognition fit for purpose [34,35] and/or to underline the importance of individual learning [36., 37., 38., 39., 40.]. We have no doubt that genetic evolution has played a role, and, given the continuing development of metacognition in late childhood and adolescence [13,20,41,42], that learning is crucial. Indeed, recent studies of human infants suggest that they may have a core, genetically inherited capacity for implicit metacognition [26,43], providing a platform for the slow development of explicit metacognition through learning and experience. However, by contrast with previous work on metacognition, we suggest that a particular kind of learning – **cultural learning** – is of overriding importance.

Learning is cultural when one agent, a receiver, learns from another agent, a sender. In cultural learning, by contrast with other kinds of social learning, what the receiver learns through social interaction with the sender is similar to, and causally dependent on, what the sender knows [44]. Cultural learning often, but not always, involves teaching. The sender may intend to communicate information to the receiver, or instead involuntarily leak information that is picked up by the receiver. If metacognition is acquired through cultural learning, it may be fit for purpose not because of gene-based selection and intentional design, but also due to cultural selection – a selection process operating on variants transmitted culturally over generations of learners.

Here, we survey evidence that metacognition is acquired through cultural learning. A stronger claim would be that metacognition is made fit for purpose by cultural selection; acquired through cultural learning and rendered adaptive by a process of natural selection acting on the culturally learned variants. There is currently less evidence in support of the stronger claim, partly because it has not yet been seriously investigated. However, there is evidence of adaptively relevant variation in metacognitive ability (i.e., in the relevant phenotype) across cultural groups and societies. Given the timescales involved, this is unlikely to be the result of gene-based selection. It is therefore plausible that cultural selection has been at work, selecting the adaptive variants in the metacognitive abilities observed in different cultural groups.

**Figure 1:**
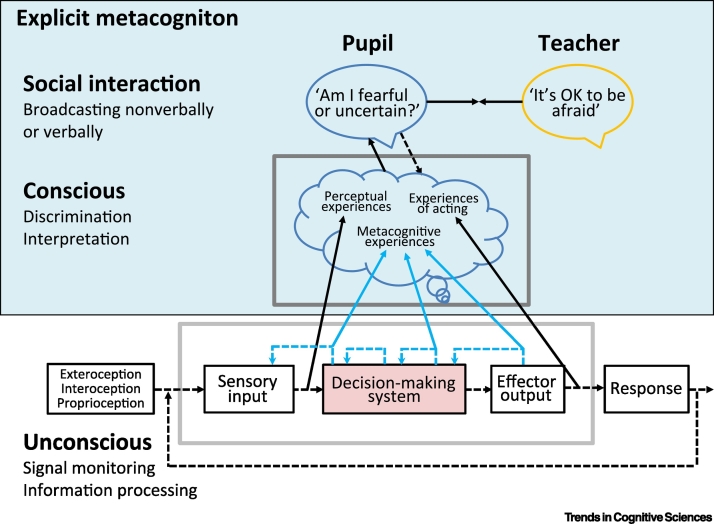
Various Signals Emerge into Consciousness and Are Available for Broadcast. Unconscious: broken blue lines – monitoring signals encapsulated within the perception–action loop. Conscious: unbroken blue lines – some monitoring signals re-represented as explicit metacognition. Solid black lines – direct experiences re-represented in consciousness. We must learn to distinguish and interpret the various signals that are re-represented in consciousness. Social interaction: we can be helped by others in this endeavour through the ability to broadcast and discuss experiences.

Current models of metacognition suggest that a range of first-order monitoring signals need to be re-represented by the metacognitive system in order to become available for the kind of intra- and suprapersonal control functions highlighted above [9,45,46] (Figure 1). Many of these first-order signals are encapsulated within the perception-action loop. For instance, if a reaching movement is subtly deviated from its trajectory by an unseen force, the person will correct the deviation without any explicit metacognitive awareness that this correction has been applied [47]. Metacognitive representations of performance are instead the result of **second-order computations** with respect to the perception–action cycle. One useful perspective on the computational problem facing metacognition is to treat it as analogous to regular perception albeit with different inputs. Just as perception is engaged with building a model of the environment from limited data, so metacognition needs to build a model of system performance using some form of inference about various cues [48]. This is consistent with the popular inferential view of how metamemory judgments are formed [49], and implies that first-order monitoring signals need to be discriminated and interpreted by the metacognitive system.

In the following, we identify three components that comprise the capacity for metacognition (Figure 1): (i) discrimination – distinguishing metacognitive feelings from one another, and from feelings that do not arise from metacognitive computations; (ii) interpretation – working out the significance of metacognitive representations, for example, whether ease of processing indicates that an object is familiar; and (iii) broadcasting – learning efficient communicative conventions for sharing metacognitive representations with other agents. As we introduce each of these components, and in the section that follows, we survey evidence that their development depends on cultural learning, and we identify opportunities to test this hypothesis further in future research.

### Discrimination

Relevant inputs for metacognition must be distinguished from one another (e.g., stimulus visibility versus decision confidence) and from interoceptive signals, including emotional states (e.g., low confidence versus fear). It would be maladaptive to share within the cognitive system, or broadcast to other agents, feelings that reflect states of the body or the world as if they represent properties of cognitive representations and processes. For instance, fear of a bear should not be mistaken for uncertainty about whether one has seen a bear. However, exactly this kind of crosstalk can be observed in laboratory experiments on metacognition. For example, when people were briefly flashed a face with a disgusted expression, their confidence in an incidental perceptual task was subtly modulated [50]. In turn, blocking noradrenaline signalling using beta blockers (potentially blunting these arousal signals) leads to an enhancement of metacognitive sensitivity [51].

Learning to discriminate metacognitive feelings from one another and from other feelings may be analogous to learning to distinguish pairs of visual [52] or olfactory [53] stimuli that were, at first, subjectively identical. Other agents who have already learned the discrimination can facilitate the process by creating environments in which different inputs are given different outcomes (e.g., rewards and punishments) or different verbal descriptions [54,55]. For example, sports coaches tell athletes that they are ‘keyed up’ or excited (high arousal) rather than unprepared (low confidence). Some children’s games give different labels to metacognitive and non-metacognitive feelings and further enhance discrimination by arranging for the child to experience no correlation or a negative correlation between them. For example, ‘peek-a-boo’ gives children alternating experiences of certainty and uncertainty – perceptual confidence is high when the adult’s face is visible and low when it is covered. These experiences are given different labels (‘Now you see me’, ‘Now you don’t’) and, crucially, feelings of surprise, resulting from both appearance and disappearance of the face, are not confounded with feelings of certainty.

### Interpretation

There is growing evidence from laboratory studies that agents learn the significance of metacognitive representations. The cultural origins hypothesis draws attention to the fact that these studies provide evidence of cultural learning. Experimenters send information to participant receivers about the significance of metacognitive feelings by structuring participants’ experience (e.g., making true statements difficult to process by reducing text/background contrast), and through verbal instruction (e.g., telling participants that true statements are often difficult to process) [56., 57., 58., 59., 60.].

Under many conditions, ease of processing a stimulus, an object or a sentence, is interpreted as indicating that the stimulus is familiar, true or attractive [61,62]. It can often be hard to discount misleading influences of fluency on our metacognitive judgments. For instance, in experiments on eyewitness memory [63], participants were asked to remember a list of faces and afterwards to indicate whether a face was previously on the list or novel, together with their confidence in this decision. Critically, half of the photographs were presented as dimly lit in the test phase, whereas half were presented as brightly lit. Increasing the brightness of the face (and, presumably, processing fluency) at test decreased accuracy in identification, but increased subjects’ confidence in their answers.

**Figure 2:**
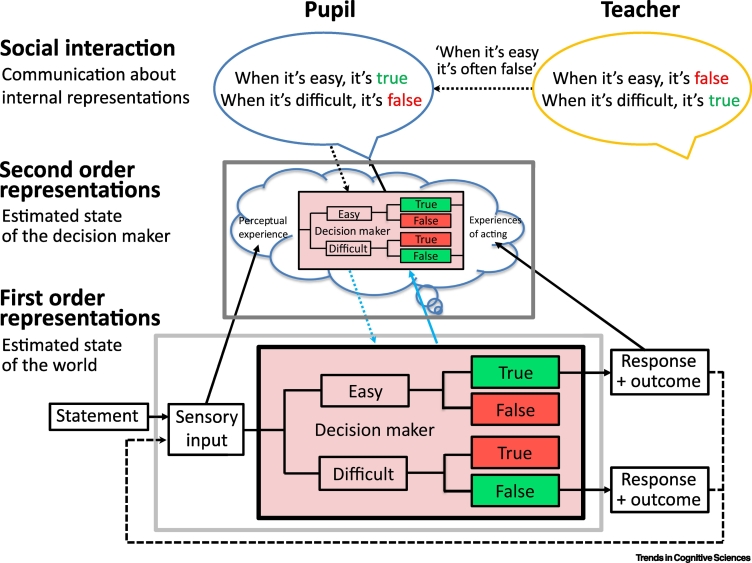
Instructions Can Change the Operation of First-Order Processes. Through the operation of second-order processes a model of decision-making at the first-order level is available for broadcasting at the second-order level. The pupil broadcasts that they believe that ‘when perception is easy, the statement is true’ (see [65]). The teacher broadcasts that the pupil’s first-order decision-making system is wrong.

These results raise the possibility that we genetically inherit a tendency to regard feelings of fluency as positive. However, when participants experience an environment in which novel stimuli are easy to process and familiar stimuli are difficult to process, they begin to interpret ease of processing as a sign that they have not seen a stimulus before [64]. Similarly, when participants are exposed to false propositions that are easy to process and true propositions that are difficult to process, they begin to interpret ease of processing as a sign of falsity [65] (Figure 2). Learning through instruction and feedback can even modulate the degree to which different components – response conflict, speed, and repetition – contribute to feelings of fluency [57]. More generally, instructions (such as ‘When it’s easy, it’s often wrong’) may change interpretation of metacognitive feelings directly, or indirectly by altering priors in subsequent feedback-based reinforcement learning (Figure 2) [57,66,67]. As an example of how instruction-based priors can shape metacognitive signals (even in the absence of explicit feedback), people told that they were clever or stupid before a working memory task showed different error-related brain responses during task performance [67].

The laboratory evidence that agents learn the significance of metacognitive representations has led to speculation about the natural environments that would support such learning [36]. Recognition that the learning is cultural should lead us to expect that, outside the laboratory, it will occur in social settings. We should expect to find learning about the context-specific significance of metacognitive representations in playgrounds, classrooms, training exercises and informal exchanges among coworkers. For example, children doing rigorous multiple-choice tests of verbal comprehension are taught that if there is an obvious answer and the question seems easy, then they should go back and read the question more carefully because they might have fallen for a lure [68]. Conversely, the overanxious person who always questions their job performance is offered strategies to discount their feelings of low confidence [69].

### Broadcasting

Broadcasting metacognitive representations to other agents for suprapersonal control involves verbal and nonverbal communication. For nonverbal communication, people automatically produce signals such as postures, action kinematics, gestures, facial expressions, and vocal qualities that convey confidence [70,71]. For example, in many English-speaking subcultures, upright posture, a serious facial expression, and vocal depth communicate assurance. These signals are in turn interpreted automatically in light of how people themselves produce these signals (e.g., two people with different speeds of movement will interpret the same action to imply different levels of confidence) [70,72]. This result indicates that receivers can derive information about senders’ confidence from nonverbal as well as verbal cues – a basic requirement for cultural learning of metacognition. Typically, nonverbal signals of confidence are not only produced but also learned without deliberation, via automatic imitation [73]. However, some institutions – such as drama schools, social skill training programmes, and debating societies at fee-paying schools – directly teach people how to act confidently. There may also be intermediate cases in which specific tools support the expression of confidence – for instance, allowing 3-year-old children to use picture-based confidence scales to indicate their confidence in perceptual decisions is sufficient for them to display above-chance metacognitive sensitivity [74].

For verbal communication, people use words that directly map onto confidence (e.g., ‘I’m sure’, ‘I think I saw it’ or ‘I dimly remember’) [27., 28., 29.] as well as signals that convey confidence nonverbally but in a controlled way (e.g., raising a hand swiftly or indicating how confident one feels on a scale from 1 to 10) [30,75]. People must solve an intricate mapping problem in order for this kind of overt communication to be effective [30,76]. In technical terms, people must map their private feelings of confidence onto public expressions of confidence in a mutually consistent manner: they must agree on not only the rank order of different expressions (e.g., that 'certain' implies higher confidence than 'sure') but also their statistical meaning (e.g., that 'sure' means that the probability that I am correct is 85% and not 75%). Laboratory studies which required groups of people to make joint decisions about ambiguous stimuli have shown that people quickly develop a common language for communicating and interpreting confidence – with better coordination leading to higher group accuracy [28,30]. More broadly, it is striking that people from the same cultural group, while not agreeing on the exact statistical meaning of different expressions of confidence, generally agree on their rank order [77].

The proposal that cultural learning shapes the gestures and words used for interpersonal broadcasting of metacognitive representations is, in many ways, the least surprising component of the cultural origins hypothesis. On reflection, few would doubt that, like other communicative conventions, we learn how to express confidence from other members of our social groups. However, here we highlight broadcasting in addition to discrimination and interpretation because the importance of communicative conventions is often overlooked in research on metacognition. For example, reports that women are less confident than men [78], and that finance professionals are more confident than average members of the population [79], are typically assumed to indicate gender and professional differences in private, intrapersonal feelings of confidence, rather than cultural differences in communicative conventions. It is, nevertheless, possible that communicative conventions for sharing metacognitive representations affect the representations themselves. For instance, there is evidence that the linguistic coding of colour [80] and space [81] can affect perception. One avenue for future research would be to test whether linguistic variation in epistemic modality (e.g., presence or absence of evidential markers [82]) affects the development of metacognition or metacognitive performance.

## Evidence of Cultural Learning

Further support for the cultural origins hypothesis, and directions for future research, comes from research on development and education, training, cultural variation, individual differences, and metacognition in non-human animals.

### Development and Education

Cultural influences on mindreading provide an analogue of the kind of developmental learning that we might also expect for metacognition. For instance, naturalistic studies have shown that individual differences [83,84] and crosscultural variation [85] in the development of mindreading covary with how much, and in what ways, mothers talk to their children about mental states. The cultural origins hypothesis predicts a similar relationship between the development of metacognition and parents’ references to confidence (e.g., ‘Do you think you can do that?’, 'Are you sure?', 'You’ve got it!’) during joint tasks (e.g., stacking bricks). This feedback would enable the infant to appropriately discriminate initially undifferentiated metacognitive experiences.

Compelling evidence of cultural learning in development also comes from intervention studies with schoolchildren in the USA and Europe. These show that metacognitive training – for example, teachers instructing pupils on goal-setting, self-questioning, and how to interpret processing dysfluency – improves text-based and mathematical learning [17,86,87] by improving metacognitive sensitivity [88] and self-reported metacognitive knowledge [17]. There is also preliminary evidence that effective teachers of literacy involuntarily leak metacognitive information that is picked up by pupils. For example, teachers’ spontaneous use of language promoting self-monitoring predicts pupils’ metacognitive awareness and independent use of reading strategies [89].

### Training

Training experiments with children and adults complement naturalistic studies in home and school settings. Previous research has shown that instruction and exposure to new contingencies can make people reinterpret feelings of fluency as signs that stimuli are unfamiliar or unattractive, and that propositions are untrue [56., 57., 58., 59., 60.] (see earlier). This evidence of reversal learning could be extended by testing the durability and context-specificity of the reversal effects; establishing whether they extend to other metacognitive feelings, such as ‘urge-to-err’ [90]; and comparing the power of overt and covert social inputs to learning – that is, feedback given by another visible agent or arranged by the experimenter behind the scenes.

The cultural origins hypothesis proposes that metacognition has been a target of cultural selection, implying that people learn from others to perform psychological operations (e.g., to discriminate metacognitive feelings), not merely to perform specific overt behaviours (e.g., to play peek-a-boo with their children). This is supported by evidence that children who have been taught an inferential skill by an adult do not replicate the adult’s teaching behaviour (e.g., use of eye contact and gestures) when they teach the skill to others [91], and by evidence that metacognitive training generalises across task domains. Carpenter and colleagues [37] showed that social feedback on confidence judgements in a perceptual task improves metacognitive calibration in both perceptual and memory tasks, indicating that what is learned through metacognitive training can be used in settings where different overt behaviours are required. Such transfer effects may in turn be mediated by the existence of domain-general metacognitive resources [92., 93., 94., 95.]. This training-and-transfer procedure could be adapted to test the cultural selection hypothesis more directly by asking people given metacognitive training on a perceptual task to collaborate with naïve participants performing a memory task [25]. If this collaboration enhanced the metacognitive sensitivity of the naïve participants, the second generation, it would provide yet stronger evidence that metacognitive operations rather than overt behaviours are culturally learned.

Further evidence of cultural training of metacognition comes from research in the humanities, social sciences and natural sciences showing that Buddhist practices are designed to, and are effective in, promoting metacognitive sensitivity (Box 2).

Box 2 **Meditation and Metacognition**

Practices that promote cultural learning of metacognition are common in many traditions, including Christian and Judaic, but they are especially prominent in Buddhism, where they have been culturally evolving for more than 2500 years.

Mindfulness (sati in Pali, smrti in Sanskrit) is central to Buddhist practice and has been closely associated with metacognition by scholars of Buddhism [131] and by western psychologists [132]. Contrary to the emphasis of many who have brought mindfulness practice to western audiences for the treatment of stress and depression, mindfulness is not necessarily nonjudgemental. Rather, in the Buddhist tradition, it involves the kind of evaluation, monitoring and control of cognitive activities characteristic of explicit metacognition [131]:

‘When mindfulness arises, sire, it reminds one of the states together with their counterparts that are wholesome and unwholesome, blameable and blameless . . . Thus, sire, mindfulness has reminding as its characteristic. . . . When mindfulness arises, sire, it examines the courses of the beneficial and unbeneficial states thus: ‘These states are beneficial; these states are unbeneficial; these states are helpful; these states are unhelpful.’ Then the one who practises yoga removes the unbeneficial states and takes hold of the beneficial states . . . Thus, sire, mindfulness has taking hold as its characteristic. [Milindapañha, 37-8]’

Experimental evidence that mindfulness-based meditation improves metacognitive sensitivity comes from both expertise and training studies. People who have chosen to practice mindfulness show greater metacognitive sensitivity in a tactile perception task than nonmeditators, and number of hours of practice (1–15 000) predict metacognitive sensitivity [133]. Similarly, long-term practitioners of mindfulness meditation are quicker than nonmediators to detect their own intention to move [134,135]. In a training study where participants were randomly chosen to receive training in mindfulness meditation or advice on nutrition, the meditation group subsequently showed greater metacognitive sensitivity in a recognition memory task [136]. Studies comparing the effects of training in different meditation practices suggest that, with an extended training period, practices designed to promote metacognition (thought-observing mindfulness), are more effective than other practices (body-focussed mindfulness) in enhancing metacognition [38,137].

Alt-text: Box 2

### Cultural Variation

There is marked cultural variation both in metacognitive bias [96] and kinds of selective social learning that may depend on metacognition [25,97,98]. For example, students from western, individualistic cultures (USA, Australia, and New Zealand) express more confidence in their decisions than students from East Asian, group-oriented cultures (Japan, Hong Kong, and Taiwan) [99], and brain imaging suggests this variation is not due solely to norms regulating the expression of confidence. The medial prefrontal cortex, a focal area for metacognition [100], is more strongly activated during self-assessment tasks in westerners than in people from less-individualistic cultures [101,102], and self-referential thoughts differentially activate hubs of the resting-state network in relation to individualistic versus collectivistic traits [103]. To our knowledge, there is no comparable research on cultural variation in metacognitive sensitivity. However, given the evidence of cultural variation in metacognitive bias, and the substantial individual variation in metacognitive sensitivity that is found within cultures [104,105], we would also expect there to be variation in metacognitive sensitivity across cultures. Crosscultural studies directly testing this prediction of the cultural origins hypothesis, by carefully quantifying and separating metacognitive bias and sensitivity, are a priority for future research.

### Individual Differences

While twin studies have indicated a genetic component in the development of metacognitive bias [106], metacognitive sensitivity is yet to be investigated using the twin method. Guided by the cultural origins hypothesis, research on individual differences in metacognitive sensitivity would include twin studies assessing the magnitude of any genetic contribution and whether it depends on a social-cognitive **endophenotype**. For example, children who are genetically predisposed to attend more closely to other agents may be better able to acquire metacognitive skill through cultural learning.

More broadly, the cultural origins hypothesis calls for research relating individual differences in metacognitive sensitivity to social skills, social experience, and education. Emulating a recent study relating metacognition to mental health [107], one approach would be to correlate metacognitive sensitivity with social and demographic indices in a large-scale online study of a general population sample. This approach would be complemented by laboratory tests. For example, if metacognitive skill is acquired by cultural learning, one would expect individual differences in imitation and mindreading [108], which are important in cultural learning, to predict individual differences in metacognitive sensitivity [109., 110., 111.]. More specifically, the cultural origins hypothesis makes a critical prediction: if metacognition has been a target of cultural selection, one would expect people with greater metacognitive sensitivity to be more effective teachers of metacognitive skills. For example, if I collaborate with someone who has high metacognitive sensitivity, in a task where confidence estimates are exchanged [27], my own sensitivity should increase more than if my collaborator has low metacognitive sensitivity.

### Non-human Animals

Given that non-human animals lack language and other cognitive resources involved in cultural learning, such as mindreading [112] and teaching [113], the cultural origins hypothesis is consistent with analyses suggesting that simple associative learning mechanisms (e.g., model-free reinforcement learning [114]) is sufficient to explain the metacognitive achievements of animals [115,116]. It is, however, notoriously difficult to assess from behavioural data alone whether metacognition in non-human animals is the product of explicit processes, or whether it might be accommodated by implicit (or first-order) computations [117]. The finding that metacognitive sensitivity can be manipulated independently of performance by prefrontal inactivation in rodents and monkeys suggests that some form of second-order computation could be in play [118,119] – but such computations may be automatic and learnt using model-free reinforcement learning. However, if the cultural origins hypothesis for metacognition is correct, we would expect a much closer correspondence between the social and metacognitive abilities of humans than in analogues of the same abilities measured in non-human animals.

In this section we have surveyed evidence that, in humans, metacognition is shaped by a specific kind of social learning – cultural learning. Social interaction provides more than motivation for the development of metacognitive skills, and information about the circumstances in which they should be used [43,120,121]. It also transmits specific metacognitive skills from experts to novices, creating the conditions necessary for cultural selection. At present, the most compelling evidence comes from research on development, education and training. Previous research on cultural variation, individual differences, and non-human animals is also consistent with cultural learning, and we have suggested strategies for further testing the cultural origins hypothesis in each of these areas of study.

## Concluding Remarks

We have suggested that cultural learning enables human agents to discriminate, interpret, and broadcast metacognitive representations. It is possible that cultural learning also plays a yet deeper role in making metacognition fit for purpose. It may bring together decision and evaluation processes to create an architecture capable of assessing the rightness of cognitive representations [48]. Alternatively, cultural learning might enable such an architecture – already developed for mindreading [109,122., 123., 124.] or in parallel with mindreading [125] – to process new kinds of input; signals from inside rather than outside the thinker, which bear on the reliability and validity of the thinker’s cognitive processes [126]. We have not pursued these possibilities because, at present, there is no empirical evidence bearing on them directly. By contrast, we have argued that education and training studies already show that cultural learning supports discrimination, interpretation, and broadcasting of metacognitive representations, and that our cultural origins hypothesis could be tested further through a programme of research in psychology and cognitive neuroscience examining development, individual differences and cultural variation in metacognitive sensitivity (see Outstanding Questions).

Metacognition is important in education, mental health, and public life. It improves learning in schools [86., 87., 88., 89.,^127^]; regulates anxiety, depression, and compulsion [107]; promotes effective leadership [128,129]; and encourages moderation in political and religious debate [22]. Metacognition is an essential ingredient not only of our capacity to know ourselves, but to know ourselves together; to make decisions in groups that are better informed, fairer, and more reasonable than the decisions that each of us can make alone. The cultural origins hypothesis suggests that the intrapersonal and suprapersonal functions of metacognition are made possible primarily by cultural learning and are adaptive in part due to cultural selection. Metacognition is tuned for social interaction by social interaction.

Outstanding Questions What is the nature and extent of cultural variation in metacognitive sensitivity? Is there cultural variation in the way confidence and error signals are computed from first order cues?

To what degree is metacognitive sensitivity genetically heritable? Is this underwritten by a social–cognitive endophenotype, such as social attention?

Are people with high metacognitive sensitivity better teachers of metacognition than people with lower metacognitive sensitivity?

What kinds of games and routines support the development of metacognition?

Do games and conversation have differential effects? For example, are games especially effective in promoting discrimination, while conversation enhances interpretation and broadcasting?

What kind of experience or training would improve metacognitive sensitivity in adulthood? Does this kind of training enhance the quality of group decision-making?

Alt-text: Outstanding Questions

## Glossary

**Conscious**: content that is globally available – that is, represented in a system and format so that it can be used, without further processing, by a wide range of cognitive processes such as planning, verbal report and storage in episodic memory.
**Cultural learning**: when a trait T (or a close variant) is acquired by learning from others who have T. Cultural learning typically involves language, teaching, imitation, or mindreading; that is, a process specialised for high fidelity transfer of information.
**Cultural selection**: increase in frequency of a trait that is transmitted by cultural learning. The increase may be because the trait is culturally transmitted more often than alternatives (transmission bias), or because the trait helps individuals to survive or reproduce.
**Endophenotype**: a genetically transmitted cause of a collection of behavioural traits. An endophenotype is useful when it shows greater heritability than the behavioural traits it typically causes.
**Explicit metacognition**: metacognition based on conscious representations in working memory, thus sensitive to interference by concurrent cognitive load.
**Gene-based selection**: increase in frequency, due to natural selection, of a trait that is inherited by transmission of genes from parents to offspring.
**Intentional design**: the capacity for designing items with the intention of fulfilling a purpose. Extends to cognitive strategies and hence their underlying mechanisms.
**Metacognition**: representation or evaluation of a cognitive state or process.
**Metacognitive bias**: the difference between reported confidence and accuracy. A subject can show metacognitive bias while demonstrating good metacognitive sensitivity (e.g., if they are systematically over- or underconfident).
**Metacognitive sensitivity**: the extent to which a subject’s confidence reports differentiate between correct and incorrect decisions – that is, the correlation between confidence and accuracy.
**Mindreading**: the ability to understand the thoughts of others, and their feelings and other mental states.
**Second-order computation**: a computation of confidence in a decision that is computed by a separate system and considers factors beyond the information used to take the decision itself [48]. Explicit metacognition is typically second order.
